# Supplementary material for: Probiotic Limosilactobacillus reuteri DSM 17938 Changes Foxp3 Deficiency-Induced Dyslipidemia and Chronic Hepatitis in Mice
Source: Nutrients. 2024 Feb 12;16(4):511. doi: 10.3390/nu16040511 (PMC10892585; doi:10.3390/nu16040511)
Supplement: Supplementary file 1 [file nutrients-16-00511-s001.zip › nutrients-2827132-supplementary.pdf]

Supplementary Table S1. PCR Primers for genes associated with lipid metabolism.

Gene Name	Forward primer	Reverse primer
Ppara	ACAAGGCCTCAGGGTACCA	GCCGAAAGAAGCCCTTACAG
Acox1	GAGCCTTTGGACCTTCACTTGG	CGCATAAGTGCCCGTGATCT
Cpt1a	GGACTCCGCTCGCTCATTC	GAGATCGATGCCATCAGGGG
Cpt2	TGTGAGCGGAAGATCCCAAC	GCTTTCCAACCCGATCTCCT
Ppargc1a	CTCTCAGTAAGGGGCTGGTTG	TTGGCTGCACATGTCCCAAG
Acot1	GGAGGTTGGGGAAAGGTACAAA	AAACTCCATTCCCAGCCCTT
Acot3	TGAGCAGTTGCCCTTGCTTTT	ATGGGCAGGGAGTTGGTGTT
Srebf1	GGGCAAGTACACAGGAGGAC	AGATCTCTGCCAGTGTTGCC
Fasn	AAGCAGGCACACACAATGGA	AGTGTTCGTTCCTCGGAGTG
Vldlr	CATGAACTCGTCCAGCCGTC	GCTGGCAGGCAGAGATATTCA
Cd36	GCCAAGCTATTGCGACATGAT	CAGATCCGAACACAGCGTAGA
Slc27a2	ATCGTGGTTGGGGCTACTTTAG	TTTGGTTTCTGCGGTGTGTTG
Slc27a5	GAGGGCAATGTGGGCTTAATG	AGGCTCTGCTGTCTCTATGTC

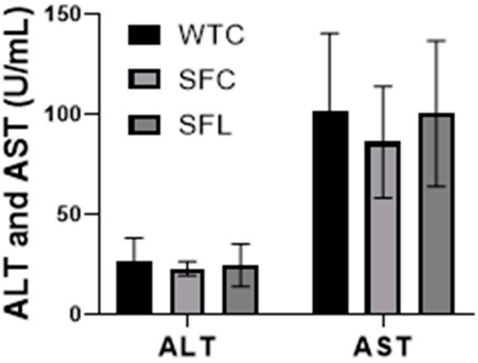

Supplementary Figure S1. Normal plasma ALT and AST levels in SF mice.
